# Supplementary material for: Prevalence and risk factors of developing cardiac arrhythmia in patients presenting to the emergency department with electrical injuries
Source: PLoS One. 2025 Dec 9;20(12):e0337812. doi: 10.1371/journal.pone.0337812 (PMC12688141; doi:10.1371/journal.pone.0337812)
Supplement: Attachment — Submitted filename: RESPONSES TO REVEIWERS.docx [file pone.0337812.s002.docx]

| **RESPONSES TO REVEIWERS** | |
| --- | --- |
| **Responses to Reviewer-1** | |
| General Evaluation: Your study focuses on the factors influencing the development of arrhythmias in patients with electrical injuries who are transported to the emergency department by prehospital medical services. Identifying these factors is valuable for patient monitoring and preparedness for early intervention. The topic is relevant and has the potential to contribute meaningfully to the literature. I would like to congratulate you on addressing this important issue. | Thank you for your valuable comment. |
| Abstract: The abstract is generally clear and appropriately reflects the overall content of the study. | Thank you for your valuable comment. |
| Introduction: The importance of the topic is well emphasized. However, the existing gap in the literature should be stated more explicitly. The originality of the study should be articulated clearly in one or two sentences. | In our study, we explained the aim of the study in more detail with the following sentences: “In this study, we aimed to investigate the prevalence of cardiac arrhythmias and the factors associated with the development of arrhythmia in patients presenting to the emergency department (ED) after an electric shock, in order to help ED physicians assess these patients and take the necessary precautions. In this way, emergency physicians and pre-hospital medical care providers will be able to identify patients at risk of developing cardiac arrhythmias at an early stage and will be informed about the need for closer and monitored observation.”. |
| Materials and Methods: Ethics committee approval is mentioned in the text, but the approval number and date are not provided. Please include this information. You have indicated that the patients were transported by prehospital medical services. However, outpatient admissions are not listed among your exclusion criteria. Please add them to the exclusion criteria. | We added the ethics committee information to the methodology section as follows: "Ethical approval from the Bilkent City Hospital Ethics Committee was obtained prior to the study (Number: TABED 2-24-326 and Date: 26.06.2024).".  Thank you for your valuable comment. We added this information as follows:” Patients were excluded if they had a history of known rhythm disorders, coronary artery disease, were younger than 18 years of age, were pregnant, did not have a routine blood test results or electrocardiogram (ECG), or if their records were inaccessible, patients who present to the hospital on their own initiative.” to the methodology section. |
| Results: The resolution of the graph in Figure 1 is low. The visual materials should be revised in higher resolution. | Thank you for your valuable comment. We revised the resolution of Figure 1. |
| Discussion: The findings are interpreted appropriately; however, in the comparison with the literature, please also address and compare any of your remaining results that were not discussed. | Thank you for your valuable comments. We have revised the discussion section to cover all of our data. |
| Conclusion: You argue that your findings may help to “prevent unnecessary hospital admissions” but this statement is somewhat overstated. The study is primarily relevant to the medical community and is not aimed at patient education. This statement should therefore be removed. | Thank you for your valuable comment. We revised the sentence as follows: “This approach will ensure that high-risk patients are not overlooked.”. |
| Language: The English used throughout the manuscript is accurate and sufficiently clear. | Thank you for your valuable comment. |
| **Responses to Reviewer-2** | |
| Abstract: The abstract is well-written, clear, and appropriately focused on the study’s objectives. It is satisfactory in its current form. | Thank you for your valuable comment. |
| Introduction: I believe you should elaborate further on how your study contributes to the existing literature and what specific gap it aims to address. | In our study, we explained the aim of the study in more detail with the following sentences: “In this study, we aimed to investigate the prevalence of cardiac arrhythmias and the factors associated with the development of arrhythmia in patients presenting to the emergency department (ED) after an electric shock, in order to help ED physicians assess these patients and take the necessary precautions. In this way, emergency physicians and pre-hospital medical care providers will be able to identify patients at risk of developing cardiac arrhythmias at an early stage and will be informed about the need for closer and monitored observation.”. |
| Materials and Methods: The name of the ethics committee and the date of ethical approval should be added.  Please clarify the rationale for defining an electric voltage above 500 as “high.” It would be appropriate to cite a reference supporting this threshold. Who evaluated the ECGs, and were the detected arrhythmias consulted with the cardiology department? Please specify. | We added the ethics committee information to the methodology section as follows: "Ethical approval from the Bilkent City Hospital Ethics Committee was obtained prior to the study (Number: TABED 2-24-326 and Date: 26.06.2024).".  In our study, we classified electrical injuries into low- and high-voltage categories in accordance with the literature. We also added the reference, and included the following statement in the methodology section: “Electrical accidents with 1000 volts and above were classified as high-voltage events [12].”.  Our study is a retrospective analysis. Patients who developed arrhythmias during follow-up were first evaluated by the emergency physician, and those requiring further assessment were subsequently consulted to cardiology. For patients who received a cardiology consultation, the electrocardiograms (ECGs) were interpreted by a cardiology specialist. In the absence of a cardiology consultation, the ECGs were evaluated based on the interpretation made by the emergency physician responsible for the patient’s care. For patients without any documented ECG interpretation, the ECGs were reviewed by two emergency medicine specialists participating in the study, each with at least ten years of clinical experience. We added this information to the methodology section. |
| Results: You categorized comorbidities as DM, HTN, and asthma. Were there no other comorbidities? If none were present, please indicate this explicitly in Table 1. | Thank you for valuable comment. We revised Table 1 and added the necessary information. |
| Discussion: In the discussion section, there is no comparison with the literature regarding some variables presented in Table 1 (e.g., time to admission). Please include such comparisons or consider removing these variables from the table | Thank you for your valuable comments. We have revised the discussion section to cover all of our data. |
| References: Please check whether your references are up to date. | Thank you for your valuable comment. |
| Tables: All tables should follow a consistent format. Please revise accordingly. | Thank you for your valuable comment. We revised our tables for the consistent format. |
| Figures: Figure 1 is not clearly readable; its quality should be improved. | Thank you for your valuable comment. We revised Figure 1 quality. |
| Language: The manuscript does not require language editing. The writing is clear and sufficiently fluent. | Thank you for your valuable comment. |
